# Aqua­dioxidobis(pentane-2,4-dionato)uranium(VI) pyrazine solvate

**DOI:** 10.1107/S1600536808009021

**Published:** 2008-04-16

**Authors:** Takeshi Kawasaki, Takafumi Kitazawa

**Affiliations:** a Department of Chemistry, Faculty of Science, Toho University, 2-2-1 Miyama, Funabashi, Chiba 274-8510, Japan; b Research Center for Materials with Integrated Properties, Toho University, Miyama, Funabashi, Chiba 274-8510, Japan

## Abstract

The asymmetric unit of the title compound, [U(C_5_H_7_O_2_)_2_O_2_(H_2_O)]·C_4_H_4_N_2_, contains one [UO_2_(acac)_2_(H_2_O)] (where acac is acetyl­acetonate) and two half-mol­ecules of pyrazine. It exhibits a UO_7_ penta­gonal-bipyramidal coordination geometry about the U^VI^ atom, involving two bidentate acetyl­acetonate ions and one water mol­ecule. The N atoms of the pyrazine mol­ecules are not coordinated to the U^VI^ atom, and are connected with the aqua O atom by hydrogen bonds. This results in a zigzag chain arrangement along the [10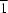] direction.

## Related literature

For related structures, see: Alcock *et al.* (1984 ▶, 1987 ▶); Alcock & Flanders (1987 ▶); Borkowski & Cahill (2004 ▶); Huuskonen *et al.* (2007 ▶); Kannan *et al.* (2001 ▶); Takao & Ikeda (2008 ▶).
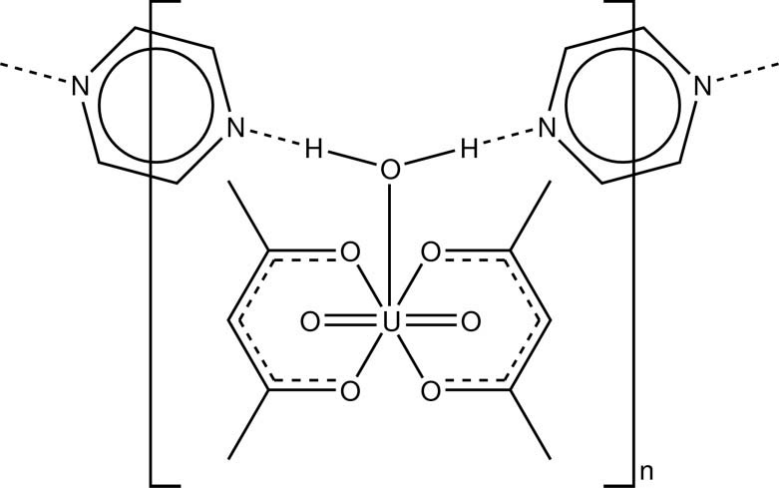


## Experimental

### 

#### Crystal data

[U(C_5_H_7_O_2_)_2_O_2_(H_2_O)]·C_4_H_4_N_2_
*M* *_r_* = 566.35
Triclinic, 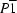

*a* = 8.186 (3) Å
*b* = 8.398 (3) Å
*c* = 13.663 (4) Å
α = 88.162 (7)°
β = 82.111 (6)°
γ = 86.130 (6)°
*V* = 928.0 (5) Å^3^
*Z* = 2
Mo *K*α radiation
μ = 8.78 mm^−1^
*T* = 299 K
0.22 × 0.14 × 0.06 mm

#### Data collection

Bruker SMART CCD area-detector diffractometer
Absorption correction: multi-scan (*SADABS*; Sheldrick, 1996 ▶) *T*_min_ = 0.236, *T*_max_ = 0.590
6928 measured reflections
4526 independent reflections
3841 reflections with *I* > 2σ(*I*)
*R*_int_ = 0.024

#### Refinement

*R*[*F* ^2^ > 2σ(*F* ^2^)] = 0.031
*wR*(*F* ^2^) = 0.078
*S* = 1.04
4526 reflections
229 parameters
2 restraints
H atoms treated by a mixture of independent and constrained refinement
Δρ_max_ = 1.63 e Å^−3^
Δρ_min_ = −1.27 e Å^−3^

### 

Data collection: *SMART* (Bruker, 2001 ▶); cell refinement: *SAINT* (Bruker, 2001 ▶); data reduction: *SAINT*; program(s) used to solve structure: *SHELXS97* (Sheldrick, 2008 ▶); program(s) used to refine structure: *SHELXL97* (Sheldrick, 2008 ▶); molecular graphics: *SHELXTL* (Sheldrick, 2008 ▶) and *CrystalMaker* (*CrystalMaker*, 2007 ▶); software used to prepare material for publication: *SHELXTL*.

## Supplementary Material

Crystal structure: contains datablocks I, global. DOI: 10.1107/S1600536808009021/hk2447sup1.cif

Structure factors: contains datablocks I. DOI: 10.1107/S1600536808009021/hk2447Isup2.hkl

Additional supplementary materials:  crystallographic information; 3D view; checkCIF report

## Figures and Tables

**Table:** 

U1—O1	1.777 (3)
U1—O2	1.774 (3)
U1—O3	2.352 (4)
U1—O4	2.348 (4)
U1—O5	2.361 (4)
U1—O6	2.353 (3)
U1—O7	2.409 (4)

**Table:** 

O1—U1—O2	178.98 (14)
O1—U1—O3	89.43 (18)
O1—U1—O4	90.62 (17)
O1—U1—O5	89.85 (18)
O1—U1—O6	91.40 (17)
O1—U1—O7	90.01 (16)
O2—U1—O3	90.37 (17)
O2—U1—O4	90.26 (16)
O2—U1—O5	89.75 (17)
O2—U1—O6	89.35 (16)
O2—U1—O7	88.97 (15)
O3—U1—O4	70.89 (13)
O3—U1—O5	145.58 (15)
O3—U1—O6	143.98 (14)
O3—U1—O7	72.72 (13)
O4—U1—O5	143.53 (13)
O4—U1—O6	73.10 (12)
O4—U1—O7	143.59 (13)
O5—U1—O6	70.43 (13)
O5—U1—O7	72.87 (13)
O6—U1—O7	143.27 (13)

**Table 2:** Hydrogen-bond geometry (Å, °)

*D*—H⋯*A*	*D*—H	H⋯*A*	*D*⋯*A*	*D*—H⋯*A*
O7—H21⋯N1	0.86 (4)	1.94 (2)	2.752 (5)	160 (5)
O7—H22⋯N2	0.85 (4)	1.96 (2)	2.778 (6)	161 (5)

**Fig. 1.**
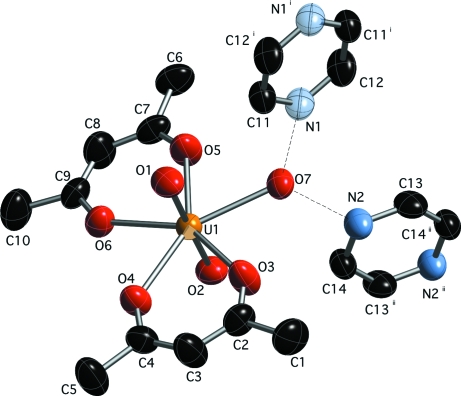
Molecular structure of (I). Displacement ellipsoids are drawn at the 50% probablity level. Hydrogen bonds are shown as dashed lines [symmetry codes: (i) -x + 1, -y + 2, -z; (ii) -x, -y + 2, -z + 1]. H atoms not involved in hydrogen bondings have been omitted for clarity.

**Fig. 2.**
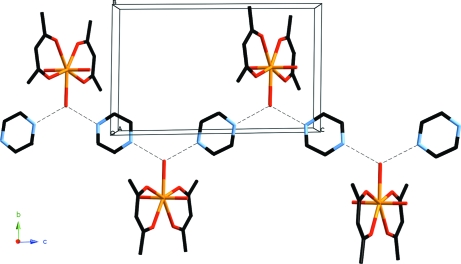
Zigzag chain arragnement formed by O7 of the [UO2(acac)2(H2O)] molecules and the pyrazine molecules in (I). Dashed lines indicate the OH···N hydrogen bonds between neighboring O7 and the pyrazine molecules. H atoms not involved in hydrogen bondings have been omitted for clarity.

## supplementary crystallographic information

### Comment

Actinide chemistry has strong relationship with the reprocessing of nuclear fuels and treatment of actinide wastes in the backend chemistry for the nuclear power plants which operate everyday. The fundamental investigation of the bonding and structure of uranium complexes provides important information on the field of backend chemistry. Various uranyl(VI) complexes with β-diketonate have been reported; examples are [UO_2_(acac)_2_(H_2_O)] (Alcock, & Flanders, 1987), [UO_2_(acac)_2_(py)] (Alcock *et al.*, 1984; Alcock *et al.*, 1987), [UO_2_(tta)_2_(H_2_O)](H_2_O)_2_(dibenzo-18, crown-6) (Kannan *et al.*, 2001), [UO_2_(acac)_2_(dmf)] (Huuskonen *et al.*, 2007), and [UO_2_(dbm)_2_(EtOH)] (Takao & Ikeda, 2008). We report herein the synthesis and crystal structure of a new uranyl(VI) acetyl- acetonate complex of formula [UO_2_(acac)_2_(H_2_O)](pz) (I) (where acac is acetylacetonate and pz is pyrazine).

The asymmetric unit of the title compound, (I), (Fig. 1), contains one [UO_2_(acac)_2_(H_2_O)] and two-halves of pyrazine molecules. The coordination geometry of the U1 atom has a UO_7_ pentagonal-bipyramidal coordination; two uranyl oxygen atoms (O1 and O2) at the axial positions, and the remaining five O atoms from the two chelating acac ligands (O3, O4, O5 and O6) and one H_2_O molecule (O7) in the equatorial plane (Table 1). The O1—U1—O2 angle is 178.98 (14) °. The deviations of the O atoms of the acac and of the H_2_O from the equatorial plane (O3, O4, O5, O6 and O7) are within 0.02 Å. The U1—O_acac_ bond lengths are longer than the U1—O_uranyl_ distances and are shorter than the U1—O_aqua_ distance. The U1—O7 [2.409 (4) Å] bond is shorter than the *U*^VI^—O_aqua_ [2.489 (8) Å] bond of [UO_2_(acac)_2_(H_2_O)] (Alcock, & Flanders, 1987), but similar to the *U*^VI^—O_aqua_ [2.396 (5) Å] bond of {[UO_2_(C_9_H_4_O_6_)- (H_2_O)].H_2_O} (Borkowski & Cahill, 2004) and the *U*^VI^—O_aqua_ [2.419 (5) Å] bond of [UO_2_(tta)_2_(H_2_O)](H_2_O)_2_(dibenzo-18,crown-6) (Kannan *et al.*, 2001).

The nitrogen atoms of the pyrazine molecules are not coordinated to the U1, and are connected with O7 atom of the H_2_O in the [UO_2_(acac)_2_(H_2_O)] molecule by the hydrogen bonds (Table 2). This results in a zigzag chain arrangement of along the [1 0 - 1] direction (Fig. 2). The dihedral angle between the two pyrazine rings is 13.9 (3)°.

### Experimental

To the acetonitrile solution (10 ml) containing UO_2_(NO_3_)_2_.6H_2_O (0.5 mmol) was added acetylacetone (3.0 mmol) and pyrazine (3.0 mmol) in acetonitrile (5 ml). After the solvent evaporated slowly at room temperature for a few days, orange crystals of (I) were obtained.

### Refinement

H atoms (for H_2_O) were located in difference syntheses and refined isotropically by applying restrains on O-H bonds [O-H = 0.852 (10) and 0.849 (10) Å; U_iso_(H) = 0.062 (16) and 0.069 (18) Å^2^]. The remaining H atoms were positioned geometrically, with C-H = 0.93 Å (for CH) and 0.96 Å (for CH_3_) and constrained to ride on their parent atoms with U_iso_(H) = xU_eq_(C), where x = 1.5 for CH_3_ H and x = 1.2 for CH H atoms.

### Crystal data

**Table:**

[U(C_5_H_7_O_2_)_2_O_2_(H_2_O)]·C_4_H_4_N_2_	*Z* = 2
*M**_r_* = 566.35	*F*_000_ = 532
Triclinic, *P*1	*D*_x_ = 2.027 Mg m^−^^3^
Hall symbol: -P 1	Mo *K*α radiation λ = 0.71073 Å
*a* = 8.186 (3) Å	Cell parameters from 2961 reflections
*b* = 8.398 (3) Å	θ = 2.4–28.2º
*c* = 13.663 (4) Å	µ = 8.78 mm^−^^1^
α = 88.162 (7)º	*T* = 299 K
β = 82.111 (6)º	Plate, orange
γ = 86.130 (6)º	0.22 × 0.14 × 0.06 mm
*V* = 928.0 (5) Å^3^	

### Data collection

**Table:**

Bruker CCD area-detector diffractometer	4526 independent reflections
Radiation source: fine-focus sealed tube	3841 reflections with *I* > 2σ(*I*)
Monochromator: graphite	*R*_int_ = 0.024
Detector resolution: 8.366 pixels mm^-1^	θ_max_ = 28.3º
*T* = 299 K	θ_min_ = 1.5º
φ and ω scans	*h* = −10→10
Absorption correction: multi-scan(SADABS; Sheldrick, 1996)	*k* = −11→9
*T*_min_ = 0.236, *T*_max_ = 0.590	*l* = −18→11
6928 measured reflections	

### Refinement

**Table:**

Refinement on *F*^2^	Secondary atom site location: difference Fourier map
Least-squares matrix: full	Hydrogen site location: inferred from neighbouring sites
*R*[*F*^2^ > 2σ(*F*^2^)] = 0.031	H atoms treated by a mixture of independent and constrained refinement
*wR*(*F*^2^) = 0.078	*w* = 1/[σ^2^(*F*_o_^2^) + (0.0402*P*)^2^ + 0.5065*P*] where *P* = (*F*_o_^2^ + 2*F*_c_^2^)/3
*S* = 1.04	(Δ/σ)_max_ = 0.001
4526 reflections	Δρ_max_ = 1.63 e Å^−^^3^
229 parameters	Δρ_min_ = −1.27 e Å^−^^3^
2 restraints	Extinction correction: none
Primary atom site location: structure-invariant direct methods	

### Special details

**Table:**

Geometry. All e.s.d.'s (except the e.s.d. in the dihedral angle between two l.s. planes) are estimated using the full covariance matrix. The cell e.s.d.'s are taken into account individually in the estimation of e.s.d.'s in distances, angles and torsion angles; correlations between e.s.d.'s in cell parameters are only used when they are defined by crystal symmetry. An approximate (isotropic) treatment of cell e.s.d.'s is used for estimating e.s.d.'s involving l.s. planes.
Refinement. Refinement of F^2^ against ALL reflections. The weighted R-factor wR and goodness of fit S are based on F^2^, conventional R-factors R are based on F, with F set to zero for negative F^2^. The threshold expression of F^2^ > 2sigma(F^2^) is used only for calculating R-factors(gt) etc. and is not relevant to the choice of reflections for refinement. R-factors based on F^2^ are statistically about twice as large as those based on F, and R- factors based on ALL data will be even larger.

### Fractional atomic coordinates and isotropic or equivalent isotropic displacement parameters (Å^2^)

**Table:**

	*x*	*y*	*z*	*U*_iso_*/*U*_eq_	
U1	0.259453 (17)	0.495967 (19)	0.247346 (9)	0.03890 (8)	
O1	0.3817 (5)	0.4969 (5)	0.1293 (3)	0.0596 (10)	
O2	0.1377 (5)	0.4988 (5)	0.3652 (3)	0.0546 (9)	
O3	0.0299 (5)	0.5981 (5)	0.1735 (3)	0.0700 (11)	
O4	0.1164 (4)	0.2826 (4)	0.2022 (3)	0.0573 (9)	
O5	0.4908 (5)	0.5598 (5)	0.3208 (3)	0.0688 (11)	
O6	0.4016 (4)	0.2596 (4)	0.2952 (2)	0.0549 (8)	
O7	0.2572 (4)	0.7828 (4)	0.2495 (2)	0.0501 (8)	
N1	0.4115 (6)	0.9337 (6)	0.0839 (3)	0.0577 (12)	
N2	0.0840 (6)	0.9322 (6)	0.4142 (3)	0.0557 (11)	
C1	−0.2101 (7)	0.6668 (8)	0.0993 (5)	0.0789 (19)	
H1A	−0.2914	0.7004	0.1534	0.118*	
H1B	−0.2637	0.6207	0.0497	0.118*	
H1C	−0.1531	0.7573	0.0713	0.118*	
C2	−0.0877 (7)	0.5442 (8)	0.1361 (4)	0.0565 (14)	
C3	−0.1090 (8)	0.3840 (8)	0.1297 (5)	0.0764 (19)	
H3	−0.1992	0.3558	0.1010	0.092*	
C4	−0.0086 (6)	0.2633 (7)	0.1619 (4)	0.0547 (12)	
C5	−0.0468 (9)	0.0931 (8)	0.1488 (6)	0.088 (2)	
H5A	0.0435	0.0399	0.1075	0.133*	
H5B	−0.1456	0.0915	0.1184	0.133*	
H5C	−0.0625	0.0393	0.2121	0.133*	
C6	0.7166 (7)	0.5944 (8)	0.4048 (4)	0.0721 (17)	
H6A	0.8071	0.6143	0.3543	0.108*	
H6B	0.7580	0.5415	0.4605	0.108*	
H6C	0.6591	0.6939	0.4249	0.108*	
C7	0.5996 (6)	0.4899 (8)	0.3648 (4)	0.0541 (14)	
C8	0.6166 (8)	0.3245 (9)	0.3785 (5)	0.0744 (18)	
H8	0.6979	0.2833	0.4152	0.089*	
C9	0.5220 (6)	0.2191 (7)	0.3416 (4)	0.0547 (12)	
C10	0.5580 (9)	0.0434 (8)	0.3557 (5)	0.086 (2)	
H10A	0.4673	−0.0008	0.3974	0.128*	
H10B	0.6569	0.0252	0.3860	0.128*	
H10C	0.5730	−0.0068	0.2927	0.128*	
C11	0.4593 (7)	0.8497 (7)	0.0033 (4)	0.0535 (13)	
H11	0.4348	0.7432	0.0030	0.064*	
C12	0.4551 (8)	1.0829 (8)	0.0803 (4)	0.0614 (15)	
H12	0.4272	1.1446	0.1362	0.074*	
C13	0.0714 (7)	1.0896 (7)	0.4270 (4)	0.0569 (14)	
H13	0.1206	1.1556	0.3771	0.068*	
C14	0.0118 (7)	0.8421 (7)	0.4888 (4)	0.0527 (12)	
H14	0.0181	0.7317	0.4833	0.063*	
H22	0.202 (6)	0.846 (5)	0.291 (3)	0.069 (18)*	
H21	0.284 (6)	0.845 (5)	0.200 (3)	0.062 (16)*	

### Atomic displacement parameters (Å^2^)

**Table:**

	*U*^11^	*U*^22^	*U*^33^	*U*^12^	*U*^13^	*U*^23^
U1	0.04099 (10)	0.04129 (13)	0.03434 (9)	−0.00227 (7)	−0.00502 (6)	−0.00051 (7)
O1	0.069 (2)	0.061 (3)	0.0440 (18)	−0.0096 (19)	0.0121 (16)	−0.0057 (17)
O2	0.058 (2)	0.056 (2)	0.0461 (17)	−0.0055 (18)	0.0052 (15)	0.0023 (17)
O3	0.077 (3)	0.050 (3)	0.091 (3)	−0.004 (2)	−0.043 (2)	0.007 (2)
O4	0.060 (2)	0.052 (2)	0.064 (2)	−0.0073 (17)	−0.0236 (16)	0.0040 (18)
O5	0.058 (2)	0.059 (3)	0.097 (3)	−0.0040 (19)	−0.036 (2)	0.000 (2)
O6	0.0554 (19)	0.053 (2)	0.0590 (19)	0.0022 (17)	−0.0185 (15)	−0.0024 (17)
O7	0.058 (2)	0.046 (2)	0.0426 (17)	0.0002 (17)	0.0050 (15)	−0.0020 (16)
N1	0.065 (3)	0.054 (3)	0.048 (2)	0.004 (2)	0.0087 (19)	0.007 (2)
N2	0.064 (3)	0.050 (3)	0.047 (2)	−0.004 (2)	0.0117 (19)	−0.004 (2)
C1	0.067 (4)	0.082 (5)	0.091 (4)	0.008 (3)	−0.031 (3)	0.015 (4)
C2	0.050 (3)	0.071 (4)	0.049 (3)	0.002 (3)	−0.016 (2)	0.007 (3)
C3	0.067 (4)	0.067 (5)	0.104 (5)	−0.012 (3)	−0.043 (3)	0.009 (4)
C4	0.055 (3)	0.056 (3)	0.056 (3)	−0.014 (2)	−0.013 (2)	0.002 (2)
C5	0.087 (4)	0.060 (4)	0.129 (6)	−0.018 (4)	−0.047 (4)	−0.003 (4)
C6	0.052 (3)	0.097 (5)	0.072 (3)	−0.011 (3)	−0.020 (3)	−0.013 (3)
C7	0.040 (2)	0.080 (4)	0.043 (2)	−0.002 (3)	−0.0084 (18)	−0.007 (3)
C8	0.065 (3)	0.073 (5)	0.091 (4)	0.008 (3)	−0.036 (3)	0.004 (4)
C9	0.044 (2)	0.062 (4)	0.056 (3)	0.011 (2)	−0.006 (2)	0.000 (2)
C10	0.090 (5)	0.065 (4)	0.105 (5)	0.016 (4)	−0.036 (4)	0.001 (4)
C11	0.067 (3)	0.043 (3)	0.048 (3)	0.000 (2)	−0.002 (2)	−0.001 (2)
C12	0.087 (4)	0.055 (4)	0.037 (2)	0.005 (3)	0.006 (2)	−0.007 (2)
C13	0.064 (3)	0.055 (4)	0.048 (3)	−0.013 (3)	0.008 (2)	0.007 (2)
C14	0.062 (3)	0.039 (3)	0.055 (3)	−0.004 (2)	0.001 (2)	−0.001 (2)

### Geometric parameters (Å, °)

**Table:**

U1—O1	1.777 (3)	C4—C5	1.505 (8)
U1—O2	1.774 (3)	C5—H5A	0.9600
U1—O3	2.352 (4)	C5—H5B	0.9600
U1—O4	2.348 (4)	C5—H5C	0.9600
U1—O5	2.361 (4)	C6—C7	1.507 (8)
U1—O6	2.353 (3)	C6—H6A	0.9600
U1—O7	2.409 (4)	C6—H6B	0.9600
O3—C2	1.265 (6)	C6—H6C	0.9600
O4—C4	1.249 (6)	C7—C8	1.396 (9)
O5—C7	1.246 (6)	C8—C9	1.366 (8)
O6—C9	1.266 (6)	C8—H8	0.9300
O7—H21	0.86 (4)	C9—C10	1.496 (8)
O7—H22	0.85 (4)	C10—H10A	0.9600
N1—C11	1.326 (7)	C10—H10B	0.9600
N1—C12	1.323 (8)	C10—H10C	0.9600
N2—C13	1.334 (8)	C11—C12^i^	1.381 (7)
N2—C14	1.344 (6)	C11—H11	0.9300
C1—C2	1.511 (8)	C12—C11^i^	1.381 (7)
C1—H1A	0.9600	C12—H12	0.9300
C1—H1B	0.9600	C13—C14^ii^	1.375 (8)
C1—H1C	0.9600	C13—H13	0.9300
C2—C3	1.376 (9)	C14—C13^ii^	1.375 (8)
C3—C4	1.361 (8)	C14—H14	0.9300
C3—H3	0.9300		
			
O1—U1—O2	178.98 (14)	O4—C4—C3	124.6 (5)
O1—U1—O3	89.43 (18)	O4—C4—C5	116.1 (5)
O1—U1—O4	90.62 (17)	C3—C4—C5	119.3 (5)
O1—U1—O5	89.85 (18)	C4—C5—H5A	109.5
O1—U1—O6	91.40 (17)	C4—C5—H5B	109.5
O1—U1—O7	90.01 (16)	H5A—C5—H5B	109.5
O2—U1—O3	90.37 (17)	C4—C5—H5C	109.5
O2—U1—O4	90.26 (16)	H5A—C5—H5C	109.5
O2—U1—O5	89.75 (17)	H5B—C5—H5C	109.5
O2—U1—O6	89.35 (16)	C7—C6—H6A	109.5
O2—U1—O7	88.97 (15)	C7—C6—H6B	109.5
O3—U1—O4	70.89 (13)	H6A—C6—H6B	109.5
O3—U1—O5	145.58 (15)	C7—C6—H6C	109.5
O3—U1—O6	143.98 (14)	H6A—C6—H6C	109.5
O3—U1—O7	72.72 (13)	H6B—C6—H6C	109.5
O4—U1—O5	143.53 (13)	O5—C7—C8	123.7 (5)
O4—U1—O6	73.10 (12)	O5—C7—C6	116.4 (6)
O4—U1—O7	143.59 (13)	C8—C7—C6	119.9 (5)
O5—U1—O6	70.43 (13)	C9—C8—C7	124.5 (5)
O5—U1—O7	72.87 (13)	C9—C8—H8	117.7
O6—U1—O7	143.27 (13)	C7—C8—H8	117.7
C2—O3—U1	137.8 (4)	O6—C9—C8	124.2 (5)
C4—O4—U1	137.9 (4)	O6—C9—C10	116.0 (5)
C7—O5—U1	138.5 (4)	C8—C9—C10	119.9 (5)
C9—O6—U1	138.2 (4)	C9—C10—H10A	109.5
U1—O7—H22	129 (4)	C9—C10—H10B	109.5
U1—O7—H21	127 (4)	H10A—C10—H10B	109.5
H22—O7—H21	102 (5)	C9—C10—H10C	109.5
C12—N1—C11	116.2 (4)	H10A—C10—H10C	109.5
C13—N2—C14	116.4 (5)	H10B—C10—H10C	109.5
C2—C1—H1A	109.5	N1—C11—C12^i^	121.4 (5)
C2—C1—H1B	109.5	N1—C11—H11	119.3
H1A—C1—H1B	109.5	C12^i^—C11—H11	119.3
C2—C1—H1C	109.5	N1—C12—C11^i^	122.4 (5)
H1A—C1—H1C	109.5	N1—C12—H12	118.8
H1B—C1—H1C	109.5	C11^i^—C12—H12	118.8
O3—C2—C3	123.5 (5)	N2—C13—C14^ii^	122.5 (5)
O3—C2—C1	116.3 (6)	N2—C13—H13	118.8
C3—C2—C1	120.2 (5)	C14^ii^—C13—H13	118.8
C4—C3—C2	125.3 (5)	N2—C14—C13^ii^	121.1 (5)
C4—C3—H3	117.3	N2—C14—H14	119.5
C2—C3—H3	117.3	C13^ii^—C14—H14	119.5

Symmetry codes: (i) −*x*+1, −*y*+2, −*z*; (ii) −*x*, −*y*+2, −*z*+1.

### Hydrogen-bond geometry (Å, °)

**Table:**

*D*—H···*A*	*D*—H	H···*A*	*D*···*A*	*D*—H···*A*
O7—H21···N1	0.86 (4)	1.94 (2)	2.752 (5)	160 (5)
O7—H22···N2	0.85 (4)	1.96 (2)	2.778 (6)	161 (5)

## References

[bb1] Alcock, N. W. & Flanders, D. J. (1987). *Acta Cryst.* C**43**, 1480–1483.

[bb2] Alcock, N. W., Flanders, D. J. & Brown, D. (1984). *J. Chem. Soc.**Dalton Trans.* pp. 679–681.

[bb3] Alcock, N. W., Flanders, D. J., Pennington, M. & Brown, D. (1987). *Acta Cryst.* C**43**, 1476–1480.

[bb4] Borkowski, L. A. & Cahill, C. L. (2004). *Acta Cryst.* E**60**, m198–m200. 10.1107/S0108270104003427 15071203

[bb5] Bruker (2001). *SMART* and *SAINT* Bruker AXS Inc., Madison, Wisconsin, USA.

[bb6] *CrystalMaker* (2007). *CrystalMaker* CrystalMaker Software Ltd., Yarnton, Oxfordshire, England.

[bb7] Huuskonen, J., Raatikainen, K. & Rissanen, K. (2007). *Acta Cryst.* E**63**, m413–m414.

[bb8] Kannan, S., Raj, S. S. & Fun, H.-K. (2001). *Polyhedron*, **20**, 2145–2150.

[bb9] Sheldrick, G. M. (1996). *SADABS* University of Göttingen, Germany.

[bb10] Sheldrick, G. M. (2008). *Acta Cryst.* A**64**, 112–122. 10.1107/S0108767307043930 18156677

[bb11] Takao, K. & Ikeda, Y. (2008). *Acta Cryst.* E**64**, m219–m220. 10.1107/S1600536807063799 PMC2915146 21200566

